# GSK-J1-loaded, hyaluronic acid-decorated metal-organic frameworks for the treatment of ovarian cancer

**DOI:** 10.3389/fphar.2022.1023719

**Published:** 2022-11-07

**Authors:** Bing Yang, Wenxu Liu, Meiying Li, Jingxin Mo

**Affiliations:** ^1^ Department of Gynecology, The Affiliated Hospital of Guilin Medical University, Guilin, China; ^2^ School of Pharmacy, Guilin Medical University, Guilin, China; ^3^ Lab of Neurology, The Affiliated Hospital of Guilin Medical University, Guilin, China; ^4^ Graduate School of Biomedical Engineering, University of New South Wales, Sydney, NSW, Australia

**Keywords:** ovarian cancer, hyaluronic acid, MOF, HER2, epigenetic modification

## Abstract

Despite intensive research, ovarian cancer has the highest mortality rates among gynecological malignancies, partly because of its rapid acquisition of chemoresistance to platinum therapy. Hence, strategies are needed to effectively treat carboplatin-resistant ovarian cancer. In this study, we designed and prepared hyaluronic acid-decorated metal-organic frameworks for the targeted delivery of GSK-J1, a JMJD3 demethylase inhibitor (HA@MOF@GSK-J1) for the synergistic treatment of carboplatin-resistant ovarian cancer. HA@MOF@GSK-J1 showed outstanding effectiveness in the inhibition of ovarian cancer *in vitro*. Furthermore, HA@MOF@GSK-J1 demonstrated higher induction of apoptosis, reduced cell motility, and diminished cell spheroids by attenuating HER2 activity through the effectual activation of H3K27 methylation in its promoter area. Finally, our *in vivo* results confirmed that HA@MOF@GSK-J1 had better treatment efficacy for carboplatin-resistant ovarian tumor xenografts. Our results highlight the potential of HA@MOF@GSK-J1 as an effective strategy to improve the treatment of carboplatin-resistant ovarian cancer.

## Introduction

Despite intensive research, ovarian cancer remains the leading cause of gynecological malignancy-related deaths (Ruth et al., 2021). Although the development of platinum and taxane chemotherapy has saved the lives of many patients with ovarian cancer (Patch et al., 2015), the rapid acquisition of chemoresistance to platinum therapy has, thus far, broadly thwarted efforts to cure patients with ovarian cancer (Zeng et al., 2019). To tackle this problem, epigenetic therapies have recently attracted attention due to their usefulness in overcoming drug resistance (Natanzon et al., 2018).

Methylating histone involves the post-synthesis modification of selected lysines on histones H3, H4, and H27 (Liu et al., 2018). Histone methylation, together with other epigenetic regulations, is associated with drug resistance in various cancers, including ovarian cancer (Curry et al., 2018). Repressive modifications (particularly H3K27me3) are globally reduced across the ovarian cancer genome (Fardi et al., 2018; Shang et al., 2019; Singh et al., 2019). The JMJD3 demethylase inhibitor, GSK-J1, showed convincing potency in suppressing ovarian cancer by restoring H3K27 methylation at the *HER2* oncogene promoter region and then suppressing its transcription (Zhang et al., 2020). However, GSK-J1 showed less efficacy in treating carboplatin-resistant ovarian cancer due to its unfavorable bioavailability and single molecular target (Duan et al., 2021). Therefore, rationally designed combinational approaches based on GSK-J1 may increase its treatment efficacy in carboplatin-resistant ovarian cancer.

Compared to traditional polymer nanoparticles, the use of inorganic nanoparticles as drug delivery vehicles has many advantages, including a large specific surface area, an easily modified surface, and facile preparation (Pugazhendhi et al., 2018). Recently, metal-organic frameworks (MOFs), a type of hybrid organic-inorganic supramolecule, have gained increasing attention as emerging nanoplatforms for biomedical applications, although at the expense of serious aggregation of nanoparticles and poor tumor targetability (Zhao et al., 2020; Tong et al., 2021).

Hyaluronic acid (HA) comprises alternating N-acetyl-D-glucosamine and glucuronic acid units (Huang and Huang, 2018a). HA containing anionic groups such as carboxylic acid and hydroxyl groups will readily bond with MOF, the surfaces of which contain abundant cationic metal ions (Li et al., 2022). Moreover, CD44, a HA receptor, is highly expressed on ovarian cancer cells, leading to CD44-mediated active targeting of tumors (Huang and Huang, 2018b). Thus, HA has gained attention in cancer-targeted drug delivery.

**SCHEME 1:**
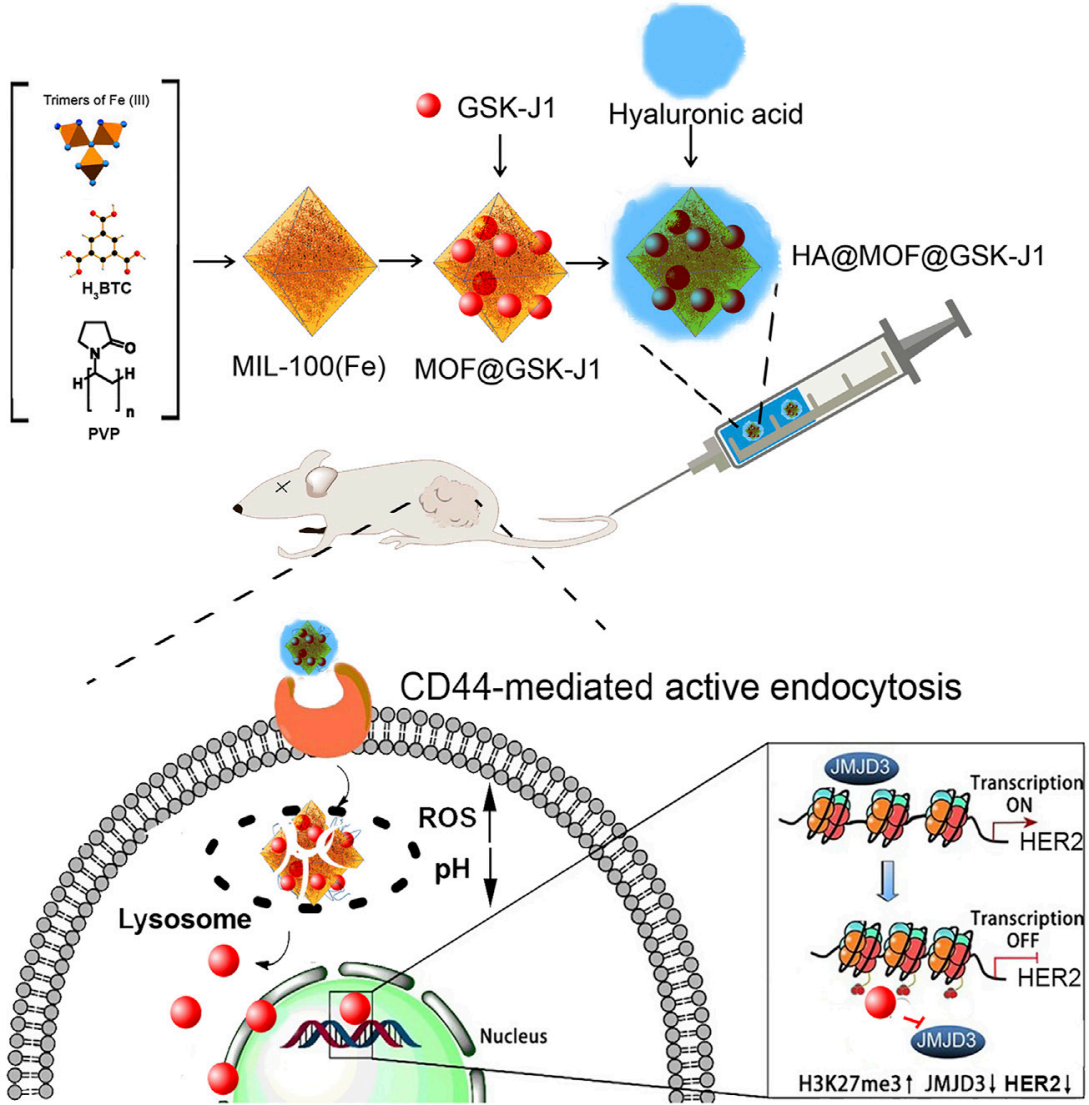
Illustration of the GSK-J1-loaded, hyaluronic acid-decorated metal-organic frameworks (HA@MOF@GSK-J1) for the targeted therapy of carboplatin-resistant ovarian tumors grafted from BALB/c nude mice *via* the epigenetic downregulation of HER2.

In this study, a GSK-J1 loaded, HA-coated MOF (HA@MOF@GSK-J1) was formulated before its physicochemical property and microstructure were evaluated. After determining the drug loading (DL) and entrapment efficiency (EE) of GSK-J1 by UV, its release profile was investigated. The cellular uptake, cytotoxicity, and possible synergism effects of the HA@MOF@GSK-J1 were explored. Then, the effects of the HA@MOF@GSK-J1 on tumor cell apoptosis, motility, and sphere formation were studied. Moreover, its epigenetic regulation on *HER2* expression was studied. Finally, the *in vivo* anti-tumor effects and preliminary toxicity were examined after tail intravenous injection of HA@MOF@GSK-J1 in nude mice bearing ovarian xenografts (Scheme 1).

## Materials and methods

### Materials

Benzene-1,3,5-tricarboxylic acid (H_3_BTC), 3-(4,5-dimethylthiazol-2-yl)-2,5-diphenyl tetrazolium bromide (MTT), and dimethyl sulfoxide (DMSO) were obtained from Aladdin Reagent Co., Ltd. (Shanghai, China). Polyvinyl pyrrolidone (PVP, MW ≈ 45–58 K) and hyaluronic acid (HA, 36 kDa), Fe(NO_3_)_3_·9H_2_O, GSK-J1, reduced glutathione (GSH) assay kit, and Cy 5.5 was purchased from Sigma-Aldrich (Shanghai, China).

### Cells

The human ovarian cancer cell line SKOV-3 was provided by the CTCC of Science (Shanghai, China) and maintained in RPMI 1640 growth medium supplemented with 10% fetal bovine serum, penicillin (100 U/mL), and streptomycin (100 μg/ml) (Gibco).

Carboplatin-resistant SKOV-3 cells (CR SKOV-3) were obtained by continuous exposure to carboplatin (Sigma-Aldrich, Shanghai, China). Starting from 18.61 μg/ml of carboplatin, the surviving cells were allowed to grow as follows: if >70% of cells survived after 72-h incubation, the SKOV-3 cells were incubated with higher concentrations of carboplatin. If >70% of the cells perished, the same concentration of carboplatin was repeated for another 72 h. This experiment lasted for approximately 6 months until carboplatin concentration reached 37.22 μg/ml. Afterward, the IC_50_ of SKOV-3 cells was evaluated. This carboplatin-resistant SKOV-3 cell line (CR SKOV-3) was used in the subsequent experiments unless otherwise indicated.

A luciferase-transfected CR SKOV-3 cell line was created from CR SKOV-3 *via* transfection of plasmid (pLenti CMV-LUC-Puro) using FuGene HD Transfection Reagent (Promega Corporation, Madison, United States). After 48 h, the cells were incubated with puromycin (0.5 μg ml^−1^) to select for transfected cells and generate a stable cell line.

### Animals

Female mice (5-week-old, BALB/c) were obtained from the Vital Laboratory Animal Center (Beijing, China). The animal experimental and housing protocol was approved by the Animal Ethics Committee of Guilin Medical University (ethics number: GLMC202003083).

### Preparation of MOF nanoparticles

MOF nanoparticles were prepared as described previously, with minor modifications (Yang et al., 2020a). First, 160 mg of Fe(NO_3_)_3_·9H_2_O and 5 mg PVP were mixed in 25 ml H_2_O and stirred for 1 hour. Then, 3 ml of 20 mg/ml H_3_BTC H_2_O solution was added to the above solution and incubated at 50°C for 1 h. The reaction solution was then centrifugated (15 min, 300 g) to collect the product and washed with ethanol three times to remove excess reactants and surfactants. The obtained MOF nanoparticles were then suspended in deionized water for further use.

### Preparation of MOF@GSK-J1 and HA@MOF@GSK-J1

GSK-J1 was loaded into the MOF by magnetic agitation of GSK-J1 in an ethanolic solution. For this, 200.0 mg of dried MOF powder was dispersed into 15 ml ethanolic solution containing 375.0 mg GSK-J1 and stirred at room temperature at 400 rpm overnight. The solution was then centrifuged (10 min, 8,000 g). The precipitate was collected and redispersed into 10 ml of distilled water before centrifugation. After two washes, the resultant product was freeze-dried to obtain the MOF@GSK-J1 nanoparticles. The above MOF@GSK-J1 nanoparticles were mixed with HA in deionized water and further sonicated for 30 min. Finally, the resulting HA@MOF@GSK-J1 NPs were rinsed three times in deionized water before being freeze-dried for further experiments.

The content of the encapsulated GSK-J1 was determined by an UV-vis spectrophotometer (Cary 50 Bio UV-Visible spectrophotometer, Varian, CA, United States) with an absorption wavelength at 278 nm in DMSO using a pre-established calibration curve (Supplementary Figure S1). The drug loading (DL) and entrapment efficiency (EE) of GSK-J1 were calculated using the following equations:

$$DL\left(\%\right)=\frac{weight\;of\;GSK-J1\;in\;nanoparticles}{weight\;of\;GSK-J1\;loaded\;nanoparticles}\times 100\%$$

$$EE\left(\%\right)=\frac{weight\;ofGSK-J1\;in\;nanoparticle\;pellet\;\left(\mu g\right)}{weight\;of\;GSK-J1\;in\;nanoparticle\;dispersion\;\left(\mu g\right)}\times 100\%\;$$

### Characterization of the MOF-formulated nanoparticles

The polydispersity index (PDI) and zeta potential of MOF@GSK-J1 and HA@MOF@GSK-J1 NPs were determined using a Zetasizer Nano ZS90 instrument (Malvern Instruments, Malvern, United Kingdom) at 25°C. The morphologies of MOF@GSK-J1 and HA@MOF@GSK-J1 NPs were imaged *via* transmission electron microscopy (TEM, Talos F200C, FEI, United States) with an acceleration voltage of 200 kV. The phase and crystal structures of MOF and MOF@HA NPs without GSK-J1 were examined by X-ray diffraction (XRD) patterns using a Rigaku X-ray diffractometer with Cu-Kα radiation (Rigaku, Japan). The FTIR spectra were recorded by Fourier transform-infrared spectroscopy (Perkin Elmer, United States).

### Stabilities of MOF@GSK-J1 and HA@MOF@GSK-J1 NPs

MOF@GSK-J1 and HA@MOF@GSK-J1 NPs (1 mg/ml) were maintained at 37°C with RPMI 1640 containing 10% FBS for 48 h. The polydispersity index and zeta potential values were recorded at 0, 2, 6, 12, 24, and 48 h to evaluate their stabilities.

### Evaluation of GSK-J1 release profiles and its possible mechanism

To evaluate GSK-J1 release behavior from MOF@GSK-J1 and HA@MOF@GSK-J1 NPs, a centrifugal method was performed as follows: First, MOF@GSK-J1 and HA@MOF@GSK-J1 NPs (5 mg) were added to 20 ml PBS (pH 5.5 or pH 7.4) and incubated in a shaker at 37°C at 100 rpm. Then, at different time points, the solution was centrifuged at 5,000 rpm for 5 min and 5 ml of the supernatant was removed to determine the concentration of released GSK-J1 by UV/vis spectrophotometry (DS5, Edinburgh Instruments) at 278 nm. The released Fe ions were detected by atomic absorption spectroscopy (Phoenix-986 AA, United Kingdom) at 248.3 nm. Then, the precipitate was resuspended after 3 ml of PBS was replenished to keep the total volume of the release media unchanged.

### Ability to reduce glutathione

MOF (2.73 μg/ml), GSK-J1 (0.39 μg/ml), MOF@GSK-J1 (3.12 μg/ml), or HA@MOF@GSK-J1 (3.78 μg/ml) was dispersed with pH 5.5 PBS with (mimicking low pH in lysosomes) and incubated with 10 μM GSH at 37°C, respectively. Twenty-four hours later, after centrifugation for 4 min at 3,000 rpm, the GSH concentration in the supernatant was determined by the reduced glutathione (GSH) assay kit at an absorbance at 450 nm according to the manufacturer instructions.

### Cellular uptake profiles of MOF@Cy 5.5 NPs and HA@MOF@Cy 5.5 NPs

We used confocal laser scanning microscopy (LSM710, Carl Zeiss, Germany) to observe the intracellular distributions of nanoparticles. In brief, to form 3D cell spheres, 5,000 CR SKOV-3 cells were seeded in 96 well-plates (Ultra Low Attachment Microplate, Corning) for 5 days. The medium was replaced with 1 ml of free-serum medium for each well, followed by the addition of free Cy 5.5 and an equal amount of Cy 5.5-loaded MOF nanoparticles (1 mg/g) with/without an HA covering. After incubation for 1 more day, the cells were fixed using 4% (v/v) paraformaldehyde and imaged under confocal laser scanning microscopes. The fluorescence intensity of Cy 5.5 in the cells was analyzed by flow cytometry (BD Bioscience).

### Subcellular location of HA@MOF@Cy 5.5 NPs

CR SKOV-3 cells were used for the cell imaging studies. The cells were seeded on 6-well plates and allowed to grow for 24 h. CR SKOV-3 cells were incubated with HA@MOF@Cy 5.5 for 6 h and then incubated with LysoTracker Blue before confocal imaging (LysoTracker Blue, Ex: 400 nm, Em: 460 nm; HA@MOF@Cy 5.5, Ex: 560 nm, Em: 650 nm).

### *In vitro* ROS production and GSH consumption of HA@MOF@GSK-J1 NPs in CR SKOV-3 cells

DCFH-DA staining assays and the reduced glutathione assay kit were used to retrospectively detect intracellular ROS and GSH levels. The CR SKOV-3 cells were treated with MOF (13.63 μg/ml), or GSK-J1 (1.95 μg/ml), MOF@GSK-J1 (15.58 μg/ml) and HA@MOF@GSK-J1 (18.90 μg/ml) for 3 h. After that, the cells were washed with PBS and incubated with DCFH-DA solution or glutathione assay kit for 20 min. Afterward, the cells were observed by CLSM.

### *In vitro* cytotoxicity tests

To evaluate the cytotoxicity of different treatments on CR SKOV-3 cells, after a 24 h growth in CR SKOV-3 cells, 100 μL of various concentrations of HA@MOF@GSK-J1 NPs, MOF@GSK-J1 NPs, free GSK-J1, or MOF were added. Control wells were added with 100 μL of PBS. After incubation for 48 h, 10 μL of MTT solution (5 mg/ml, Aladdin Reagent, Shanghai, China) was added to each well, the absorbance values of which were determined by using a microplate reader (Emax Precision, United States) at 570 nm. And IC_50_ was calculated using OriginPro 2019b (Originlab, Northampton, US). The combination index (CI) was calculated from the following formula and used to define synergism.

$$CI=\frac{{IC}_{50}\left(\mu M\right)\;of\;HA@MOF@GSK-J1}{{IC}_{50}\left(\mu M\right)\;of\;MOF}+\frac{{IC}_{50}\left(\mu M\right)\;of\;HA@MOF@GSK-J1}{{IC}_{50}\left(\mu M\right)\;of\;GSK-J1}$$

### Annexin V/PI apoptosis detection

Apoptosis of CR SKOV-3 cells was evaluated using an Annexin V-FITC/PI Apoptosis Detection Kit according to the manufacturer’s protocol. Briefly, CR SKOV-3 cells were plated at a density of 5 × 10^5^ cells/well into 6-well plates and placed in a 37°C, 5% CO_2_ incubator overnight. Then, MOF (13.63 μg/ml), GSK-J1 (1.95 μg/ml), MOF@GSK-J1 (15.58 μg/ml), or HA@MOF@GSK-J1 (18.90 μg/ml) were applied to treat the cancer cells. After 1 day, the cells were stained with annexin-FITC/propidium iodide for 0.5 h in the dark and analyzed by flow cytometry.

### Wound-healing migration assay

After 24 h pre-incubation until ∼90% confluent monolayers had formed, the CR SKOV-3 cells were scratched with a 20 μL pipette tip and subsequently treated with MOF (2.73 μg/ml), GSK-J1 (0.39 μg/ml), MOF@GSK-J1 (3.12 μg/ml), or HA@MOF@GSK-J1 (3.78 μg/ml) for 48 h at 37°C. Images were taken at 0 and 48 h. Wound healing analysis was performed using Image J software (NIH, United States).

### Boyden chamber assay

We assessed the ability of MOF (2.73 μg/ml), or GSK-J1 (0.39 μg/ml), MOF@GSK-J1 (3.12 μg/ml) or HA@MOF@GSK-J1 (3.78 μg/ml) against CR SKOV-3 cell invasion through transwell assays. Briefly, 20,000 cells were plated in Transwell chambers (Corning Costar, St. Louis, MO, United States) on top of the pre-coated inserts in 200 μL serum-free media. The reservoir well was filled with 10% FBS containing media with different formulations (700 μL). The plates were incubated in 5% CO_2_ at 37°C with the different treatments for 1 day. The remaining cells were then removed from the upper membrane. The number of cells on the lower membrane was counted and photographed using a microscope (5 microscopic fields/well).

### Inhibition of CR SKOV-3 tumor spheroids

To assess the anti-tumor spheroid ability for different formulations, CR SKOV-3 cells were seeded at a density of 3 × 10^3^ per well in an ultralow-attachment round-bottom 96-well plate (CAT#7007, Corning). Five days later, the cell spheroids were treated with MOF (2.73 μg/ml), GSK-J1 (0.39 μg/ml), MOF@GSK-J1 (3.12 μg/ml), or HA@MOF@GSK-J1 (3.78 μg/ml) for 3 days, respectively. The volumes of CR SKOV-3 tumor spheroids were evaluated before and after incubation. The major (*d*_max_) and minor (*d*_min_) diameters of each tumor spheroid were recorded, and the volumes (V) were calculated according to the equation:

$$V=0.5\times d_{\max}\times d_{\min}^{2}$$

### qPCR assay

RNA was extracted from cells using a Qiagen RNeasy Kit. cDNA was prepared using the iScript cDNA Synthesis Kit (Bio-Rad) according to the manufacturer’s instructions. Quantitative gene expression analysis was performed on an iCycler IQ (Bio-Rad, CA, United States) using SYBR Green Master Mix (Bio-Rad) with a primer for each of the transcripts. All reactions were duplicated. qPCR primers are listed in Supplementary Table S1.

### ChIP-qPCR analysis

The assay was performed using an EZ-Zyme Chromatin Prep Kit (Millipore), according to the manufacturer’s protocol. Anti-H3K27me3′ antibody (Cat# 9783, Cell Signal Technology) was used in the immunoprecipitation reaction with H3K27me3. The pulled DNA underwent purification and was analyzed by RT-qPCR with primers specific to the predicted binding sites on the promoter site. Immunoprecipitated DNA and whole-cell extracted DNA were also treated for reverse crosslinking using the Zymoclean PCR purification kit (Zymo). The ChIP-PCR primers are listed in Supplementary Table S1.

### Western blotting

To assess the expression levels of HER2 protein, the CR SKOV-3 cells were administered different treatments and later lysed in a modified RIPA buffer (No. P0013B, Beyotime Biotechnology, China) and quantified by BCA protein assay. Briefly, the protein samples (30 μg per lane) were loaded onto a 10% gel for sodium dodecyl sulfate-polyacrylamide gel electrophoresis, before being transferred onto poly-vinylidene fluoride (PVDF) membranes. Next, the PVDF membranes were blocked in 5% non-fat milk for 1 h and incubated with primary antibodies diluted 1:10,000 rabbit anti-HER2 (ab214275, Abcam) and 1:5,000 rabbit anti-β-actin (ab8224, Abcam) at 4°C overnight and goat anti-rabbit IgG antibody for 1 h. Finally, the membranes were exposed to a chemiluminescence substrate (Thermo Scientific) and visualized on a ChemiDoc™ MP Imaging System (Bio-Rad).

### Biodistribution of Cy5.5-labeled MOF formulations *in vivo*

To visualize the *in vivo* biodistributions, the MOF formulations were loaded on Cy5.5 as a fluorescent probe for evaluating their biodistributions. The CR SKOV-3 tumor-bearing mouse model was established by subcutaneously injecting cells (1 × 10^6^ cells/100 μL PBS/mouse) into the rear left thigh of the BALB/c mice. When the tumor volumes reached 100–300 cm^3^, 100 μL of Cy 5.5 solution or MOF@Cy5.5 and HA@MOF@Cy5.5 (Cy5.5, 2 mg/kg) were tail intravenously injected into tumor-bearing nude mice. At 1, 2, 4, 8, 12, 24, 48, 72, 96, and 120 h post-administration, the mice were observed on an IVIS Spectrum *in vivo* image system (Caliper Life sciences, MA, United States) after being anesthetized. To compare fluorescence intensity in different organs and tumors, the mice were euthanized at 24 h post-injection of different formulations. The vital organs including the heart, liver, spleen, lungs, kidneys, brain, and tumor tissue were observed on an IVIS Spectrum *in vivo* image system.

### Anti-tumor effects in the ovarian cancer xenograft model

The studies on ovarian cancer xenograft inhibition involved the subcutaneous injection of luciferase-labeled luciferase-transfected CR CR SKOV-3 cells (1 × 10^6^ cells in 100 μL PBS) into the rear right thighs of the BALB/c nude mice. On the seventh day after tumor cell injection, the mice were allocated into five groups (*n* = 5). For these five treatment groups, the doses were equivalent to 10 mg drug/kg: (group 1) sham (0.9% saline injection solution); (group 2) MOF (10 mg/kg in 0.9% saline injection solution); (group 3) GSK-J1 (10 mg/kg dissolved in 0.9% saline injection solution); (group 4) MOF@GSK-J1 (equivalent to 10 mg/kg of GSK-J1 in 0.9% saline injection solution); (group 5) HA@MOF@GSK-J1 (equivalent to 10 mg/kg of GSK-J1 in 0.9% saline injection solution). Treatments were injected through the tail vein once a week for four consecutive weeks. 200 µL of 15 mg/ml firefly D-luciferin was injected intraperitoneally and mice were anesthetized by 3% isoflurane. Tumor growth was visualized with an IVIS Spectrum *in vivo* image system.

After 4 weeks, the nude mice were euthanized. Their vital organs and tumors were then removed and fixed in 4% paraformaldehyde. The vital organs (heart, liver, spleen, lungs, kidneys, and brain) were harvested for HE staining. TUNEL assays were performed simultaneously to assess tumor cell growth and apoptosis.

### Statistical analysis

All quantitative data are expressed as means ± standard deviation (SD) unless otherwise noted. Statistical significance was tested using unpaired, two-tailed Student’s *t*-tests. *P < 0.05 was considered indicative of statistically significant differences.

## Results

### Nanoparticle characterization

**FIGURE 1:**
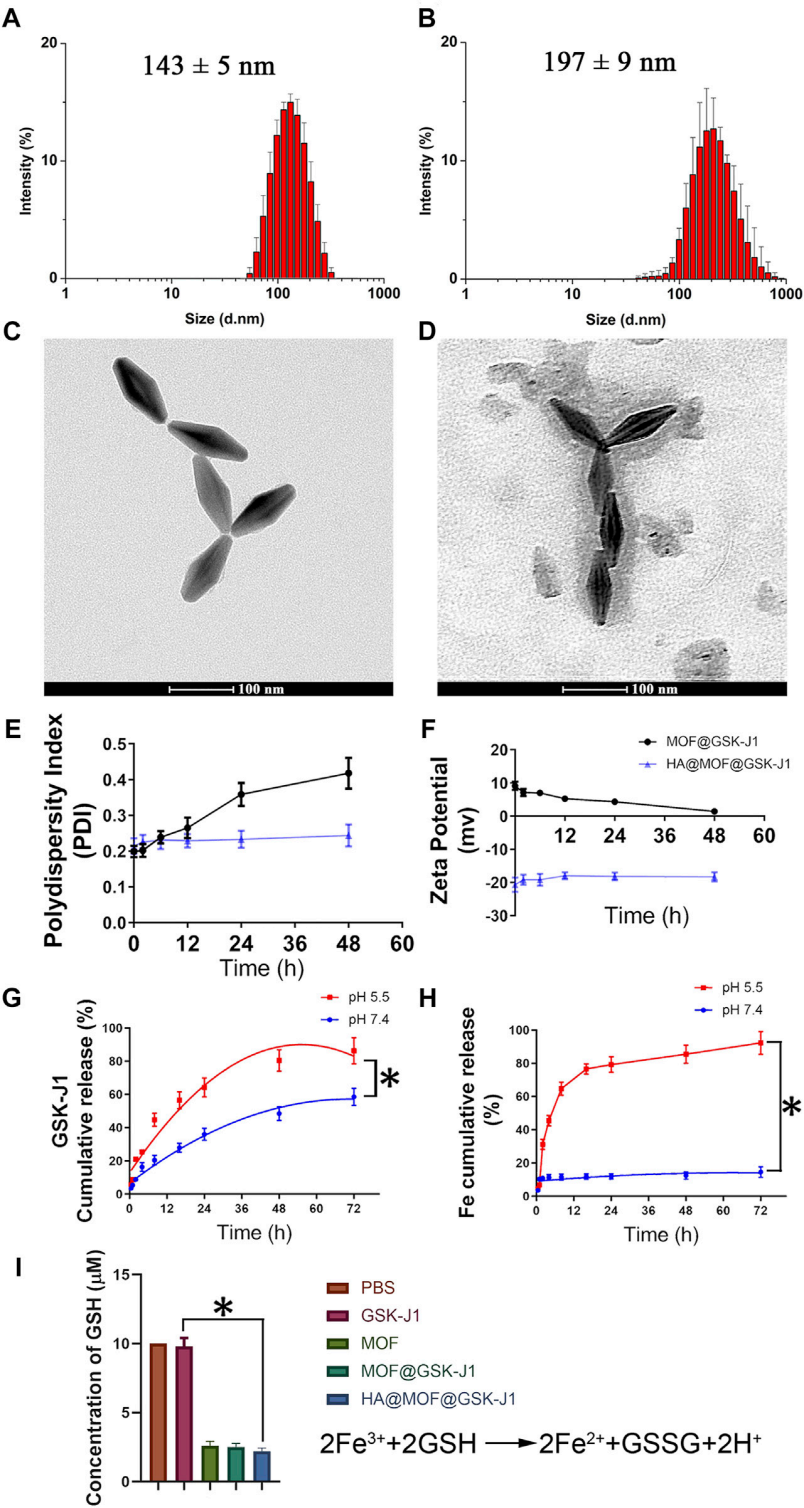
Size distributions of **(A)** MOF@GSK-J1 and **(B)** HA@MOF@GSK-J1 based on DLS. Transmission electron micrographs of **(C)** MOF@GSK-J1 NPs and **(D)** HA@MOF@GSK-J1 NPs. Scale bar: 100 nm. **(E)** Polydispersity index and **(F)** zeta potential of MOF@GSK-J1 and HA@MOF@GSK-J1 NPs after incubation in RPMI 1640 containing 10% FBS at 37°C for 48 h. **(G)** Accumulative release of MOF@GSK-J1 and HA@MOF@GSK-J1 NPs after incubation in pH 5.5 and pH 7.4 PBS at 37°C for 72 h. **(H)** Accumulative release of Fe^3+^ after incubation in pH 5.5 and pH 7.4 PBS at 37°C for 72 h **(I)** GSH concentrations after incubation in different formulations of pH 5.5 PBS at 37°C for 24 h. The results are expressed as mean ± SD, *n* = 3; *, *p* < 0.05.

Examination of the sizes, size distributions, and morphology of the different nanoparticles showed that covering MOFs with HA visibly affected the sizes (Figure 1A,B). The average particle size of the HA@MOF@GSK-J1 NPs was 197 ± 9 nm, increased from 143 ± 5 nm for MOF@GSK-J1 NPs without HA covering. As shown in the TEM images in Figures 1C,D, MOF@GSK-J1 was diamond-shaped, with narrow dispersion, while the HA@MOF@GSK-J1 NPs showed a translucent layer of HA.

We further studied the stability of MOF@GSK-J1 and the HA@MOF@GSK-J1 NPs in RPMI 1640 containing 10% FBS at room temperature by monitoring their zeta potential and PDI (Figures 1E,F). The zeta potential and PDI of HA@MOF@GSK-J1 NPs remained steady in RPMI 1640 containing 10% FBS at 37°C for at least 48 h. The PDI of MOF@GSK-J1 showed an upward trend, from 0.199 to 0.418, in the same conditions, which was related to the decreasing zeta potential from 9.13 mv to 1.42 mv after 48 h. The zeta potential showed a significant decrease for HA@MOF@GSK-J1 NPs (−20.84 ± 0.28 mV), suggesting that HA binds to the residue amino groups of MOFs with abundant -OH groups, leading to a charge-shielding effect.

Drug loading on MOF@GSK-J1 and HA@MOF@GSK-J1 NPs were 12.5 ± 3.1% and 10.3 ± 3.7%, respectively. The entrapment efficiencies (EE) of MOF@GSK-J1 and HA@MOF@GSK-J1 NPs were 89.5 ± 6.3% and 91.5 ± 7.94%, respectively. As shown in Figure 1G, the cumulative release of GSK-J1 from HA@MOF@GSK-J1 was 58.56% after incubation in pH 7.4 PBS for 72 h. However, the percentage of GSK-J1 released from the HA@MOF@GSK-J1 increased from 58.56% to 86.30% by lowering the pH from 7.4 to 5.5 after 72 h incubation. This acidic pH-responsive release behavior may be caused by the collapse of the MOF as a remarkable amount of Fe^3+^ dissociated from the MOFs at pH 5.5 (Figure 1H). As a critical intracellular antioxidant, GSH serves a protective role as an antioxidant defense. The dissociated Fe^3+^ could consume GSH and produce GSSH, thus resulting in a redox imbalance. As shown in Figure 1I, compared to the group treated with GSK-J1, the GSH concentration decreased significantly after treatment with HA@MOF@GSK-J1.

Compared to MOFs, the FT-IR spectra of HA@MOF showed bands at ∼1,598 cm^−1^ and ∼1,487 cm^−1^ peaks corresponding to the symmetrical and asymmetrical stretching peaks of νC = O. The bands at ∼1,023 cm^−1^, ∼1,082 cm^−1^, and ∼1,162 cm^−1^ correspond to typical the peaks of glycosyl in hyaluronic acid, signifying successful covering by hyaluronic acid (Supplementary Figure S2A). Supplementary Figure S2B shows the XRD patterns of the MIL-100(Fe) NPs before and after covering then with HA. All the diffraction peaks in the XRD patterns showed the same characteristic peaks as reported, indicating that the successful coating of HA did not alter the MOF crystallinity but slightly decreased the reflection intensity.

### *In vitro* cell sphere uptake

**FIGURE 2:**
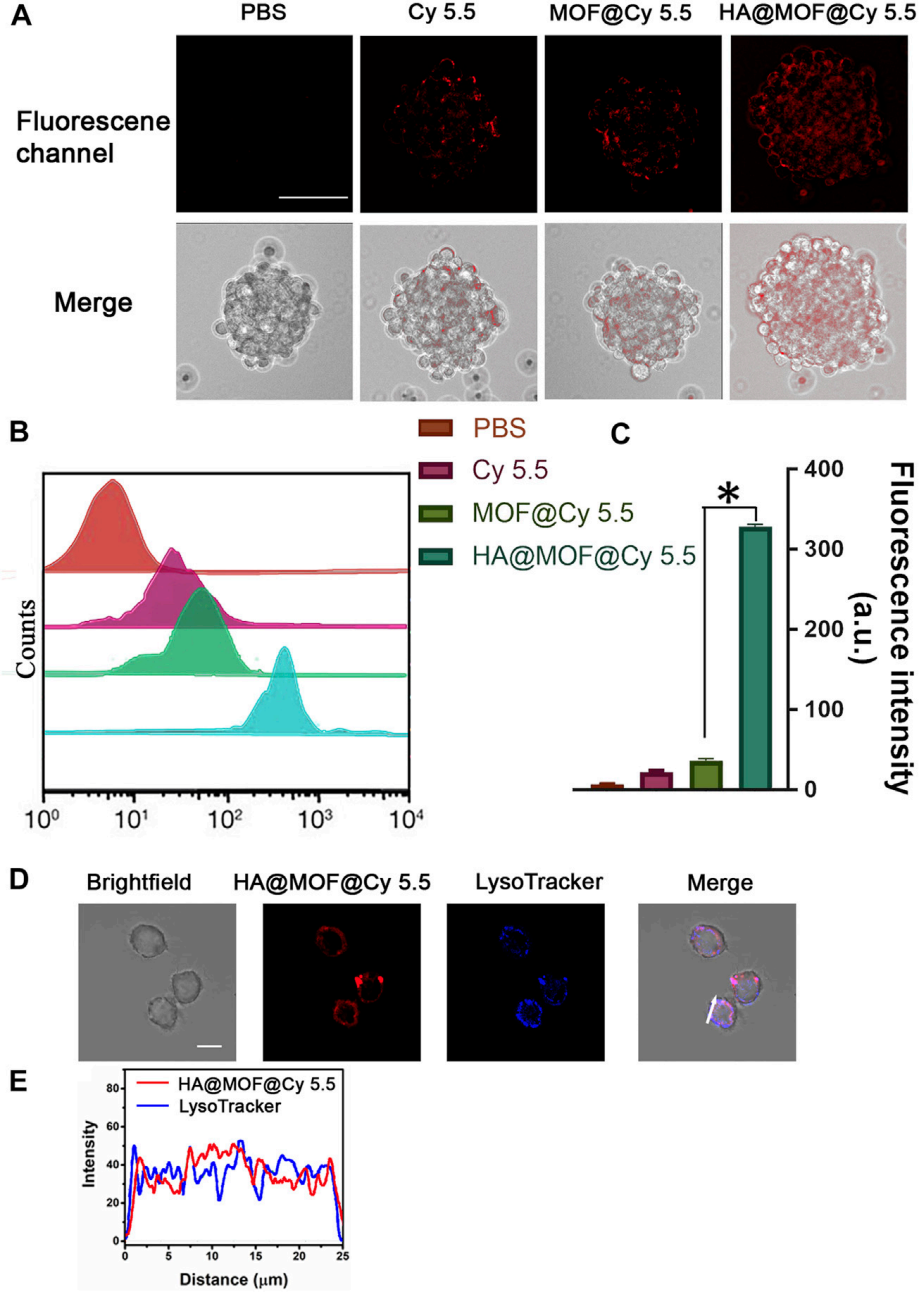
**(A)** *In vitro* cellular uptake of PBS, Cy 5.5, MOF@Cy 5.5 NPs, and HA@MOF@Cy 5.5 after incubation with CR SKOV-3 spheroids. Scale bar: 100 μm. **(B)** Fluorescence intensity in CR SKOV-3 spheroids analyzed by flow cytometer. **(C)** Histogram of Cy 5.5 fluorescence intensity in different treatment groups. Data are presented as means ± SD, *n* = 3, *, *p* < 0.05. **(D)** Co-localization of HA@MOF@Cy 5.5 with LysoTracker Blue in CR SKOV-3 cells. Scale bar: 20 μm. **(E)** Correlation plot of HA@MOF@Cy 5.5 with LysoTracker Blue in CR SKOV-3 cells (white arrow).

The cellular uptake of PBS, Cy 5.5, MOF@Cy 5.5 NPs, and HA@MOF@Cy 5.5 NPs were measured in CR SKOV-3 cell spheres. After 3 h of incubation, the cells were imaged by CLSM (Figures 2A,B) and further quantified by flow cytometry (Figures 2C,D). Compared to MOF@Cy 5.5 NPs, HA@MOF@Cy 5.5 showed an 8.99-fold higher uptake efficiency in CR SKOV-3 spheroids, demonstrating the higher uptake efficiency due to the stronger penetration ability of 3D cell spheroids, possibly facilitated by the HA coating. An additional advantage of HA coating is the selective targeting of cancer and controlled drug release by HAdase inside the cancer cells.

To evaluate the destiny of HA@MOF@Cy 5.5 after cell uptake, we performed co-localization tests in CR SKOV-3 cells with LysoTracker Blue dye. CR SKOV-3 cells were incubated with HA@MOF@Cy 5.5 and then incubated with LysoTracker Blue for confocal imaging. As shown in Figures 2D,E, after CR SKOV-3 cells were incubated with the HA@MOF@Cy 5.5 for 6 h, the coincidence degree of fluorescence signals for the red and blue channels was very good (the Pearson’s co-localization coefficient: 0.86), which suggested that HA@MOF@Cy 5.5 may experience the lysosome pathway after active endocytosis by CD44 receptors. The acidic lysosome microenvironment would then aid HA@MOF@Cy 5.5 decomposition and payload release into the cytoplasm.

### Evaluation of ROS production and GSH consumption in different formulations

**FIGURE 3:**
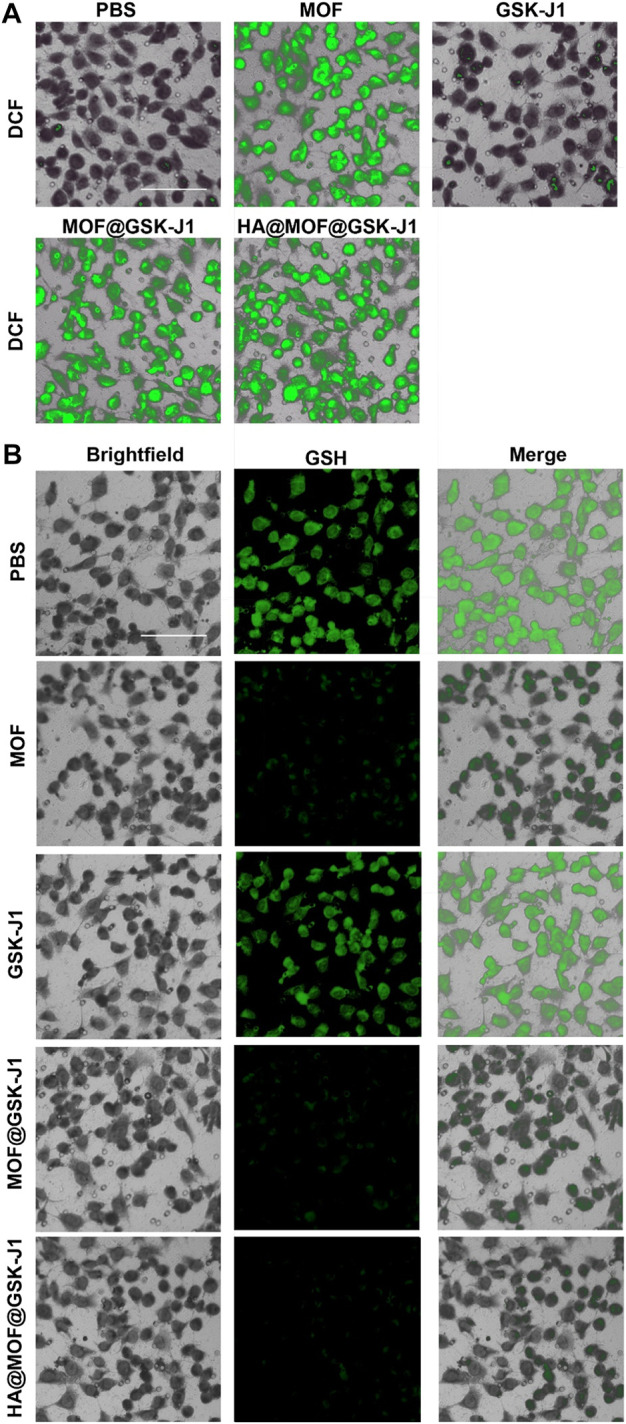
CLSM images of intracellular **(A)** ROS generation and **(B)** GSH consumption by PBS, MOF, GSK-J1, MOF@GSK-J1, and HA@MOF@GSK-J1. Scale bar: 100 μm.

Previous studies suggested that the anti-tumor mechanism of Fe^3+^-based MOFs includes the intracellular overproduction of ROS by converting H_2_O_2_ to •OH and GSH to GSSG (Li et al., 2021). To investigate ROS production of different formulations inside CR SKOV-3 cells, the production of intracellular ROS was observed after dying the cells with 2′,7′-dichlorofluorescein diacetate (DCFH-DA). Similar to the PBS group, very weak DCFH-DA fluorescence was visible inside CR SKOV-3 cells treated with GSK-J1. While a brilliant DCFH green fluorescence was observed after co-culture with MOF-based formulations like MOF, MOF@GSK-J1, and HA@MOF@GSK-J1 (Figure 3A). The capacity of HA@MOF@GSK-J1 to consume intracellular GSH was further analyzed in CR SKOV-3 cells. As shown in Figure 3B, much lower intracellular GSH levels were observed after treatment with MOF-based formulations such as MOF, MOF@GSK-J1, and HA@MOF@GSK-J1 than with PBS or GSK-J1. Effective GSH consumption together with the accumulation of intracellular ROS may provide tumor therapy by inflicting a cellular redox imbalance.

### *In vitro* synergistic antitumor effects of HA@MOF@GSK-J1

**FIGURE 4:**
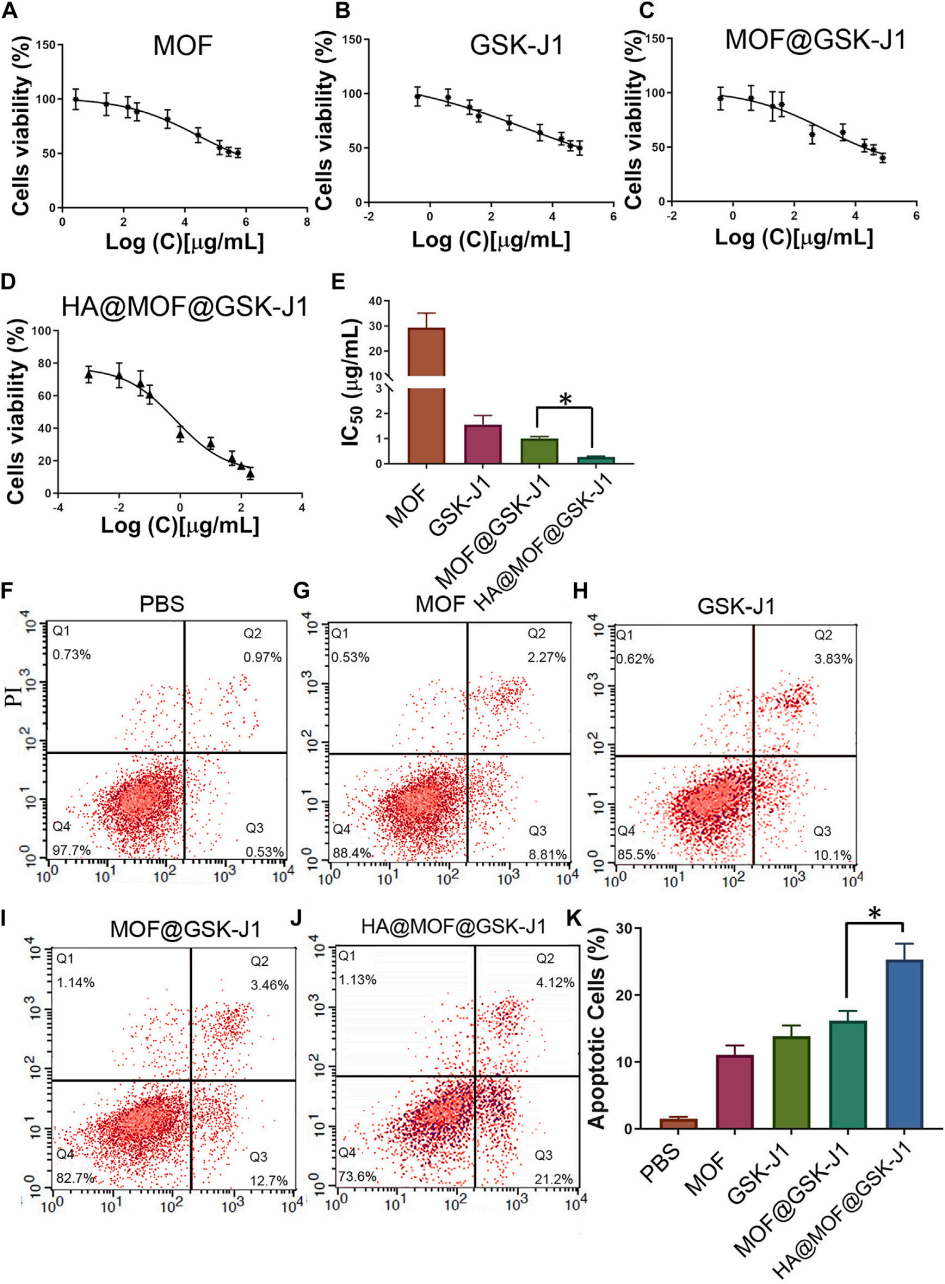
**(A–D)** Cytotoxicities of CR SKOV-3 cells treated with various concentrations of MOF, GSK-J1, MOF@GSK-J1, and HA@MOF@GSK-J1. **(E)** Histogram of cytotoxicity after different treatments. **(F–J)** CR SKOV-3 cell apoptosis determined by flow cytometry after incubation with PBS, MOF, GSK-J1, MOF@GSK-J1, and HA@MOF@GSK-J1, respectively. **(K)** Histogram of CR SKOV-3 cell apoptosis after different treatments. *n* = 3, *, *p* < 0.05.

The IC_50_ of carboplatin in CR SKOV-3 cells (4.70 ± 1.15 μg/ml) was much higher than that of primitive SKOV-3 cells (0.94 ± 0.12 μg/ml), indicating the successful establishment of the CR SKOV-3 cell line (Supplementary Figure S3). Figures 4A–E show that the IC_50_ of GSK-J1 was lower than that of the MOF. The IC_50_ values for the CR SKOV-3 cell line were 29.31 ± 5.83 μg/ml for the MOF and 1.56 ± 0.36 μg/ml for GSK-J1, respectively. Furthermore, MOF@GSK-J1 and HA@MOF@GSK-J1 NPs further significantly reduced cell viability. The IC_50_ values of the CR SKOV-3 cell line were 1.01 ± 0.07 μg/ml for MOF@GSK-J1 and 0.27 ± 0.03 μg/ml for HA@MOF@GSK-J1 NPs, respectively. The IC_50_ of HA@MOF@GSK-J1 NPs was significantly lower than those of the MOF@GSK-J1 and other treatment groups. Collectively, the HA@MOF@GSK-J1 (CI = 0.24) showed a great synergistic antitumor effect (Synergism if CI-value <0.8).

To determine the role of different nanoparticles in CR SKOV-3 cell apoptosis, we used flow cytometry to detect apoptosis. The HA@MOF@GSK-J1 NPs showed the highest apoptosis rate (25.32 ± 2.40%), while the MOF@GSK-J1 NPs, MOF, GSK-J1, and negative control showed apoptosis rates in descending order (Figures 4F–J). Together, as shown in Figure 4K, the HA coating led to a much higher apoptosis rate than those caused by the MOF, GSK-J1, or MOF@GSK-J1, respectively.

### HA@MOF@GSK-J1 suppresses cancer cell metastasis and renewal properties *in vitro*

**FIGURE 5:**
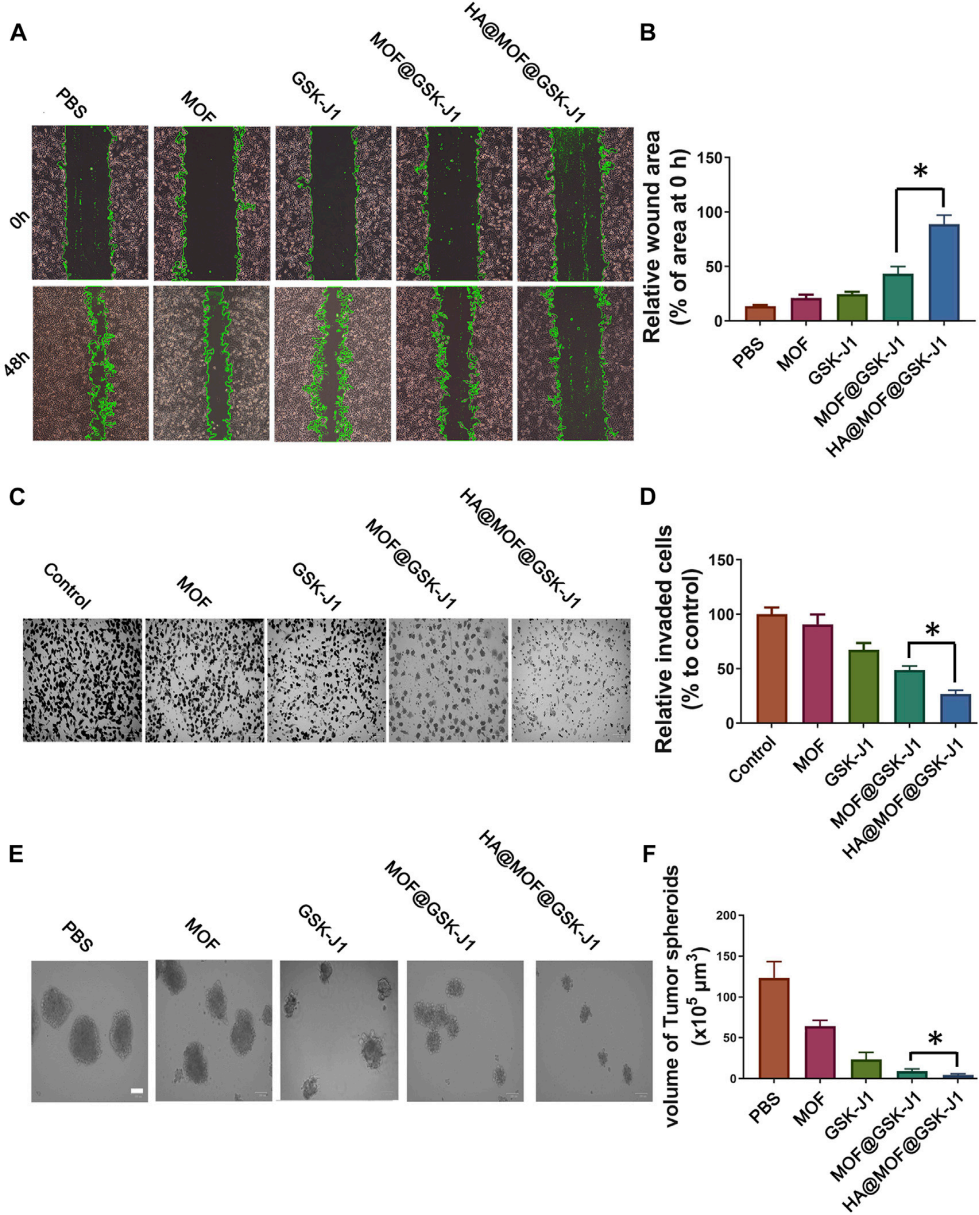
Effects of HA@MOF@GSK-J1 on CR SKOV-3 cell migration, invasion, and self-renewal. **(A)** Confluent CR SKOV-3 cells were scratched and incubated with MOF, GSK-J1, MOF@GSK-J1, and HA@MOF@GSK-J1, respectively. The area covered by the migrating cells was imaged at 0 and 48 h. **(B)** Rates of wound closure in wound scratches receiving different treatments. **(C)** CR SKOV-3 cell invasion tested by Boyden chamber assays. Media containing 0.1% FBS (−) was used as the negative control. **(D)** Quantification of migrated CR SKOV-3 cells after incubation with MOF, GSK-J1, MOF@GSK-J1, and HA@MOF@GSK-J1, respectively. Tumor suppressor effects of GSK-J1 and HA@MOF@GSK-J1 NPs were observed when CR SKOV-3 cells were cultured as tumor spheroids in 3D assay platforms. **(E)** Representative images of tumor spheroids after incubation with MOF, GSK-J1, MOF@GSK-J1, and HA@MOF@GSK-J1, respectively, for 3 days. Scale bar: 100 μm. **(F)** Tumor spheroid volumes after treatment with MOF, GSK-J1, MOF@GSK-J1, and HA@MOF@GSK-J1. Data are presented as means ± SD of three independent experiments. *p < 0.05.

The migration ability of tumor cells is a vital indicator of metastasis. Hence, we studied the inhibitory capabilities of MOF (2.73 μg/ml), GSK-J1 (0.39 μg/ml), MOF@GSK-J1 (3.12 μg/ml), and HA@MOF@GSK-J1 (3.78 μg/ml) on the horizontal and vertical migration of cancer cells. As shown in Figures 5A–D, the scratch areas remained nearly unchanged with the HA@MOF@GSK-J1 treatments. The number of migrated cells decreased significantly after HA@MOF@GSK-J1 treatment (Figure 5C). The migration inhibition rate decreased from 48.6 ± 3.9 to 26.8 ± 3.3% after incubation with HA@MOF@GSK-J1 compared to MOF@GSK-J1 (Figure 5D). These results suggested the potential antimetastatic effects of HA@MOF@GSK-J1.

We performed sphere formation assays to assess the efficiency of anti-tumor spheroids of HA@MOF@GSK-J1 for 3 days. We observed the formation of smaller cell spheres following HA@MOF@GSK-J1 treatment. The average cell sphere volume among 10 cell spheres in the HA@MOF@GSK-J1 group was much less than those in the MOF@GSK-J1 (13 cell spheres) and other treatment groups (16 cell spheres). As shown in Figures 5E,F, the tumor spheroid volume in the control group was about 123.5 ± 19.7 × 10^5^ μm^3^ on the 3rd day. The spheroid volume ratios to the control group treated with MOF, GSK-J1, MOF@GSK-J1, and HA@MOF@GSK-J1 were about 52.0%, 19.1%, 7.3%, and 3.6%, respectively. These results suggested that HA@MOF@GSK-J1 treatment enhanced anti-tumor spheroids in CR SKOV-3.

### HA@MOF@GSK-J1 reduces CR SKOV-3 cell viability by epigenetic downregulation of HER2

**FIGURE 6:**
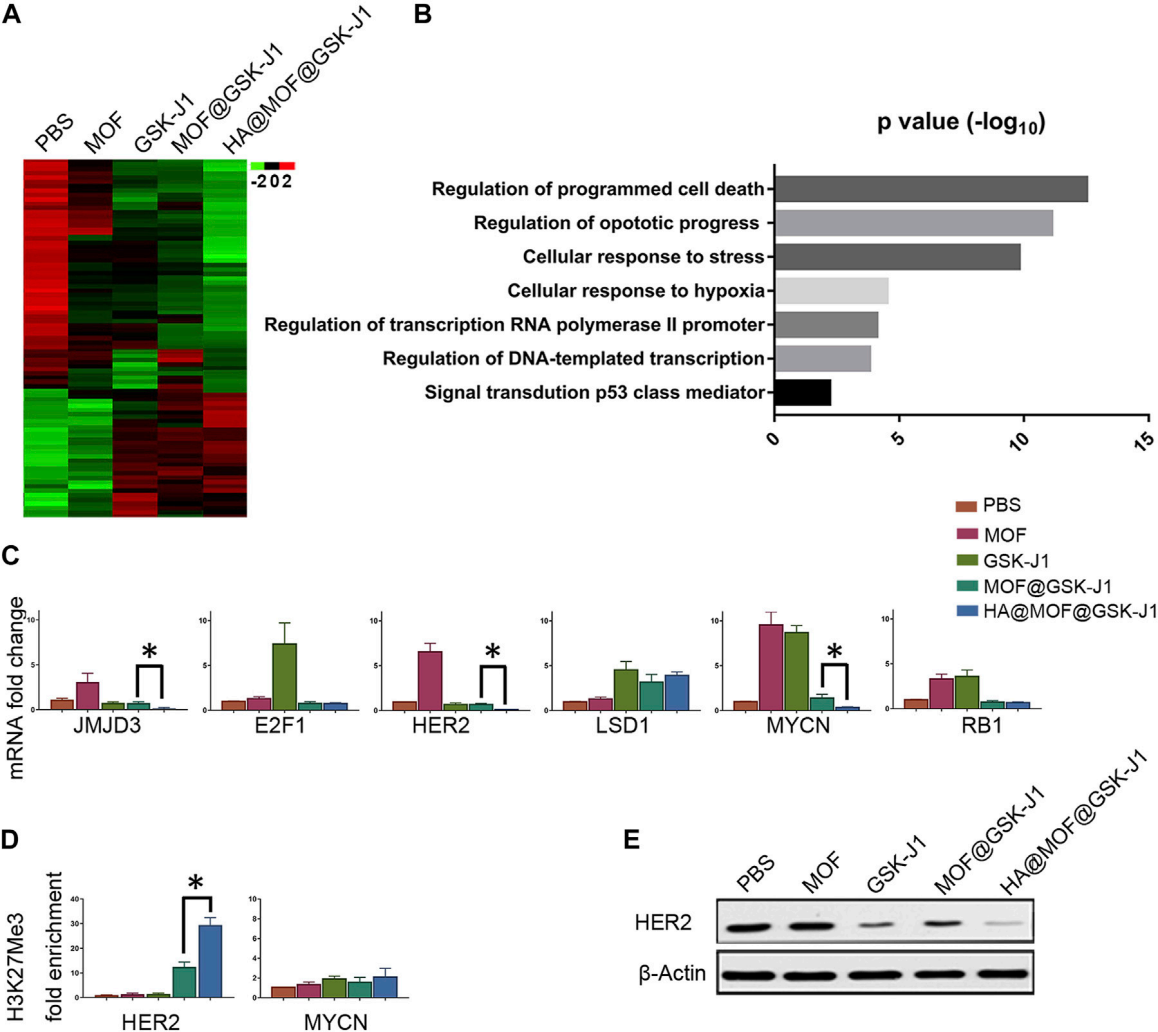
HA@MOF@GSK-J1 affects a JMJD3-mediated gene network. **(A)** Heat-map of gene expression in CR SKOV-3 cells after different treatments **(B)** KEGG analysis of the biological pathways of the genes in regulated CR SKOV-3 cells following treatment with HA@MOF@GSK-J1 compared to treatment with MOF@GSK-J1 treated. **(C)** Histograms showing the RNA fold-induction of different treatments. qPCR analysis of CR SKOV-3 cells was performed using the indicated primers after different treatments (Supplementary Table S1). Amplification of GAPDH transcripts was used to normalize the loading of each RNA sample. **(D)** ChIP-qPCR analysis of H3K27me3 mark of the HER2, and MYCN promoters in CR SKOV-3 cells treated with different formulations. **(E)** Western blot analysis of HER2 in CR SKOV-3 cells after different treatments. All results are expressed as fold-inductions (means) from three independent experiments. The bars indicate SD (*p < 0.05, Student’s t-tests).

To study the genes involved in CR SKOV-3 cell chemoresistance to platinum therapy, we performed gene expression profiling by sequencing total RNA from the cells after different treatments (Figure 6A). We identified significantly differentially expressed genes that accumulated in critical aspects of cancer function after treatment with HA@MOF@GSK-J1 (Figure 6B). Next, we used qPCR to analyze the expression of selected genes from KEGG-GO analysis after different treatments. We confirmed that HA@MOF@GSK-J1 intensely decreased *JMJD3*, *MYCN*, and *HER2* levels; these genes are involved in epigenetic regulation, metastasis, and ovarian cancer tumor stemness (Figure 6C). To determine if JMJD3, an H3K27me3-specific demethylase, directly affected the transcriptional regulation of *MYCN* and *HER2* in CR SKOV-3 cells, we used ChIP assays to assess the H3K27me3 levels in the promoter regions of these genes. HA@MOF@GSK-J1 significantly increased the binding of H3K27me3 histone3 in the *HER2* promoter region, indicating transcriptional repression. However, we did not detect changes in H3K27me3 levels at the *MYCN* promoter region (Figure 6D). We also demonstrated that HER2 protein levels were lowest after HA@MOF@GSK-J1 treatment among all treatment groups by western blotting (Figure 6E).

### HA@MOF@Cy5.5 shows enhanced accumulation in tumors

To study the targeting capability of the vehicle, Cy 5.5, MOF@Cy 5.5, and HA@MOF@Cy 5.5 were intravenously injected into the tails of CR SKOV-3 tumor-bearing nude mice and observed on an *in vivo* image system.

**FIGURE 7:**
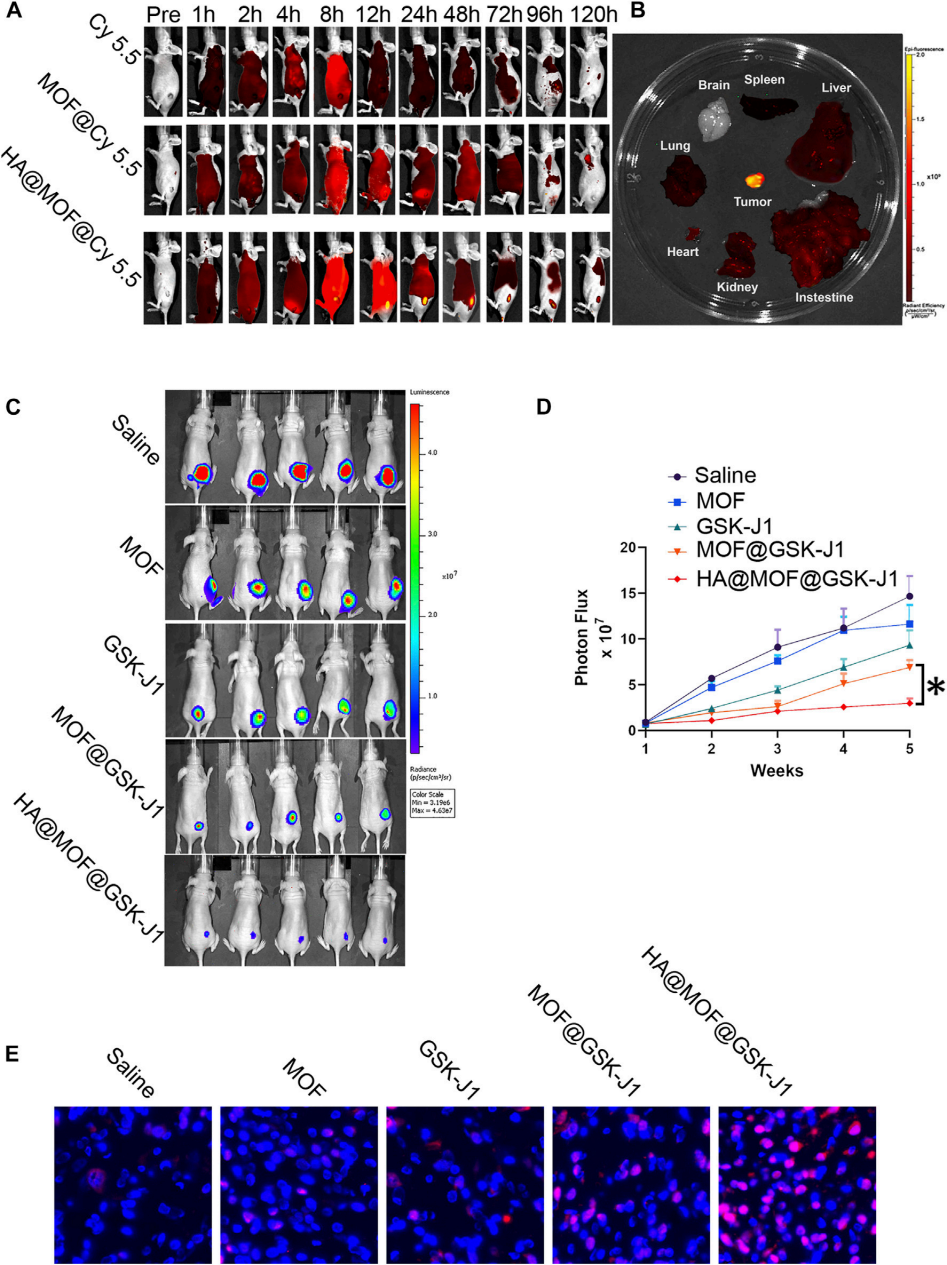
**(A)** Typical fluorescence images of CR SKOV-3 tumor-bearing mice for up to 120 h after tail intravenous injection of Cy 5.5-labeled formulations. **(B)** Typical fluorescence images of vital organs and tumor tissue dissected from CR SKOV-3 tumor-bearing mice at 24 h after tail intravenous injection of HA@MOF@Cy 5.5. **(C)** Therapeutic outcomes of mice with CR SKOV-3 xenografts. Bioluminescence imaging of mice after the indicated treatments of 10 mg/kg MOF, GSK-J1, MOF@GSK-J1, and HA@MOF@GSK-J1, respectively. **(D)** Tumor growth was monitored by bioluminescence using an *in-vivo* imaging system. Tumor size is expressed in radiance units (photons/s/cm^2^/sr). The results are presented as means ± SD, *n* = 5. *p < 0.05. **(E)** Typical TUNEL immunohistochemical staining images of the tumors following various treatments. Scale bar: 20 μm.

As shown in Figure 7A, mice treated with HA@MOF@Cy5.5 exhibited the strongest fluorescent signal at the tumor site compared to the other treatment groups for up to 120 h. The fluorescence intensity plateaued at 24 h after injection. We hypothesized that this result occurred largely due to the particular ligand of HA, which enhanced tumor targetability *via* CD44 receptor-mediated endocytosis, and the protective hydrophilic properties of HA, which increased the circulation time. As shown in Figure 7B, HA@MOF@Cy5.5 displayed much higher accumulation in tumor tissue compared to that in the vital organs. However, no fluorescence was observed in the brain, suggesting its immunity to the central nervous system.

### HA@MOF@GSK-J1 inhibits cancer cells *in vivo*

We assessed the *in vivo* efficacy of MOF, GSK-J1, MOF@GSK-J1, and HA@MOF@GSK-J1 against ovarian cancer based on the measurement of luciferase expression in *in vivo* bioluminescence imaging. The bioluminescence intensities of the tumors were monitored 4 weeks after treatment. As shown in Figures 7C,D, bioluminescence imaging revealed different therapeutic effects. Rapid tumor growth in the saline-treated control group was observed, while the tumor growth in the MOF and GSK-J1-treated groups was slightly less. In contrast to the MOF and GSK-J1-treated groups, the tumor growth in the MOF@GSK-J1-treated group was the slowest, with the lowest tumor bioluminescence values among all treatment groups.

To further investigate the anticancer mechanism of HA@MOF@GSK-J1 at the molecular level, TUNEL assays were performed to detect apoptosis. The fluorescent images demonstrated the highest apoptotic cell ratio in the HA@MOF@GSK-J1 group (Figure 7E). In contrast, the MOF and GSK-J1 groups showed the lowest ratios among the treatment groups (Figure 7E). These results indicated the potent anticancer efficacy of HA@MOF@GSK-J1.

### Preliminary toxicity evaluation

**FIGURE 8:**
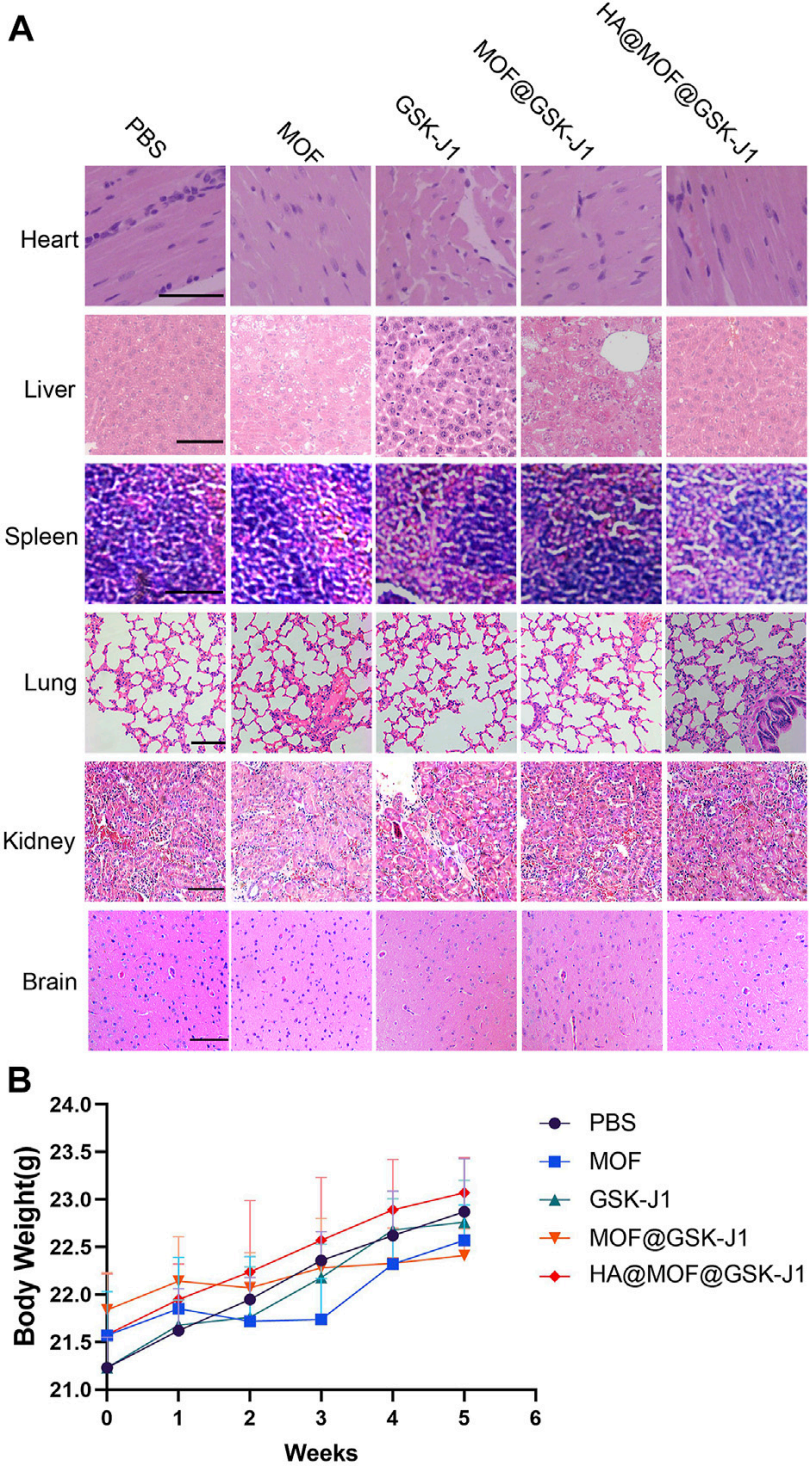
**(A)** Hematoxylin and eosin (HE) staining of the vital organs of healthy mice treated with MOF, GSK-J1, MOF@GSK-J1, and HA@MOF@GSK-J1. Scale bar: 100 μm. **(B)** Body weight changes of nude mice bearing CR SKOV-3 tumors after the intravenous injection of different formulations. *n* = 5.

Histological examinations of the major organs including the heart, liver, spleen, kidneys, lungs, and brain of the experimental animals were evaluated by hematoxylin-eosin (H&E) staining (Figure 8A). No abnormal cellular structures or systemic toxicity were observed in normal mice (without tumor xenograft) treated with MOF, GSK-J1, MOF@GSK-J1, and HA@MOF@GSK-J1. The lack of significant weight loss during treatment also confirmed the safety of these treatments (Figure 8B).

## Discussion

To explore the potential clinical utility of GSK-J1 in carboplatin-resistant ovarian cancer, we evaluated the antitumor effect of GSK-J1-loaded HA-covered MOF, in which the combination of HA@MOF@GSK-J1 was the most effective against carboplatin-resistant cells and in the ovarian animal model.

Reactive oxygen species (ROS) are highly reactive molecules derived from oxygen and produced in cells by oxidases (Wang et al., 2019). Besides free-radical and non-free radical oxygen-containing molecules, ROS can be generated from reactive nitrogen and transition metals such as iron (Fe), copper (Cu), and sulfur species (Bicalho et al., 2021). Furthermore, metal-based nanoparticles such as MOFs can also produce ROS (Xie et al., 2021).

In our study, we synthesized nanoscale MIL-100(Fe) *via* a hydrothermal route with Fe (III) salt and H_3_BTC. The metal-organic framework-Fe^3+^ (MOF) had dominant characteristics including high surface area and porosity, wide flexibility, and shape tunability (Xie et al., 2021). Evidence suggests that Fe^3+^-containing MOFs could only degrade and release ferrous iron ions in low-pH environments (pH 2.0–5.0) (Chen et al., 2022), which could be generated by the following reaction:

$${2Fe}^{3+}+2GSH\to 2{Fe}^{2+}+GSSH+2H^{+}$$

Furthermore, the ferrous iron released from MOFs may react with H_2_O_2_ to become ferric ions and release •OH radicals to damage cancer cells according to the following chemical reaction.

$$2{Fe}^{2+}+H_{2}O_{2}=2{Fe}^{3+}+2∙OH$$

Hence, as shown in Figure 3, formulations containing MOF can generate intensive ROS after uptake by CR SKOV-3 cells.

Although MOFs are considered excellent carriers for GSK-J1; however, among the challenges facing the use of MOF-based nanocarriers, their colloidal stability and the premature release of drug cargo are the most concerning (Xu et al., 2022). In MOF-based nanocarriers, premature release can occur due to the nature of the interaction between the drug and the MOF (Liu et al., 2021) Therefore, MOFs may serve as promising carriers for anti-cancer treatment only after proper evaluation.

HA comprises alternating D-glucuronic acid and N-acetylglucosamine units (Cadete and Alonso, 2016). HA, which has a high affinity for CD44 receptors, has also been used as a targeting moiety (Yang et al., 2020b). HA has excellent biocompatibility, biodegradation, and specific targeting for CD44 (Song et al., 2022). HA, with a negative charge, has good interface affinity with MOFs, whose surface charge is positive, to form a stable nanoscale shell. Moreover, HA-based surface modification may give Bio-MOF targeted anti-cancer characteristics including the promotion of aggregation on tumor sites and increased MOF uptake by CD44-positive cancer cells (Pieterse et al., 2019). Therefore, higher anticancer treatment efficacy may be achieved by using tumor-targeting ligand HA to encapsulate and stabilize MOFs.

In the present study, the protective coating of HA resulted in a more stable GSK-J1-loaded MOF with a sustainable release when tested in cell culture media (Figure 2). *In vitro* assessment of cellular uptake in CR SKOV-3 cells revealed a preferential uptake of HA@MOF@Cy 5.5 into CR SKOV-3 ovarian cancer cells overexpressing CD44 (a cell surface glycoprotein). One possible reason for this differential uptake is that the HA coating of MOFs may specifically conjugate with the CD44 on the surface of CR SKOV-3 ovarian cancer cells (Shariati et al., 2020). The *in vivo* results also confirmed that HA coating leads to the preferential accumulation of MOFs and their payloads in CR SKOV-3 ovarian cancer sites after tail vein injection of HA@MOF@Cy 5.5 (Figure 7).

Ovarian cancer is the deadliest gynecological cancer (Shahin et al., 2018). Increasing evidence suggests that acquired resistance originates from abnormal epigenetic changes to tumor suppressor genes (Oing et al., 2019) To identify possible signal molecule involved in the anti-ovarian cancer effect of HA@MOF@GSK-J1, we performed DNA microarray gene expression analysis in CR SKOV-3 cells (Figure 6A). HA@MOF@GSK-J1 treatment markedly decreased HER2 expression and affected the expression of many genes associated with cancer proliferation (Figure 6B). These findings suggest that HA@MOF@GSK-J1 may have multiple targets for its anti-ovarian cancer effects.

More specifically, the mechanism involves HA@MOF@GSK-J1-induced suppression of HER2 expression and protein levels (Figure 6C,E). Many studies have confirmed the effects of HER2 overexpression on cancer progression and poor prognosis in ovarian cancer (Satpathy et al., 2019; Cho et al., 2020; Dhritlahre and Saneja, 2021). Therefore, we analyzed epigenetic alterations in the HER2 promoter region. HA@MOF@GSK-J1 induced increased H3K27me3 (Figure 6D). Therefore, HA@MOF@GSK-J1 showed promising epigenetic suppressing effects to effectively treat ovarian cancer. These effects suggest that HA@MOF@GSK-J1 may decrease HER2 expression by increasing H3K27 methylation.

## Conclusion

The results of this study demonstrated that the combination of GSK-J1 and MOF may have synergistic effects in the treatment of carboplatin-resistant ovarian cancer because MOF increased the levels of reactive oxygen species while GSK-J1 reduced HER2 levels in cancer cells. We also found that covering MOF@GSK-J1 with hyaluronic acid improved the targeted release of GSK-J1 within ovarian tumors. Furthermore, HER2 activity may be reduced by HA@MOF@GSK-J1 through the activation of H3K27 methylation in its promoter area. Therefore, the high sensitivity of carboplatin-resistant cells to HA@MOF@GSK-J1 could be exploited to develop novel combined adjuvant therapies for this rapidly progressing and invariably lethal cancer.

## Data Availability

The original contributions presented in the study are included in the article/Supplementary Material. Further inquiries can be directed to the corresponding author.
