# Supplementary material for: GSK-J1-loaded, hyaluronic acid-decorated metal-organic frameworks for the treatment of ovarian cancer
Source: Front Pharmacol. 2022 Nov 7;13:1023719. doi: 10.3389/fphar.2022.1023719 (PMC9676248; doi:10.3389/fphar.2022.1023719)
Supplement: Supplementary file 1 [file DataSheet1.docx]

***GSK-J1 loaded hyaluronic acid decorated metal-organic frameworks to treat ovarian cancer***

Bing Yang^1^, Wenxu Liu^3^, Meiying Li^3^, Jingxin Mo^2,4,^*

*^1^* *Department of Gynecology, the Affiliated hospital of Guilin Medical University, Guilin, China,*

*^2^* *Lab of Neurology, The Affiliated Hospital of Guilin Medical University, Guilin, China,*

*^3^School of Pharmacy, Guilin Medical University, Guilin, China,*

*^4^* *Graduate School of Biomedical Engineering, University of New South Wales, Sydney, NSW, Australia.*

Correspondent E-mail:

Jingxin Mo: Jingxin.mo@hotmail.com


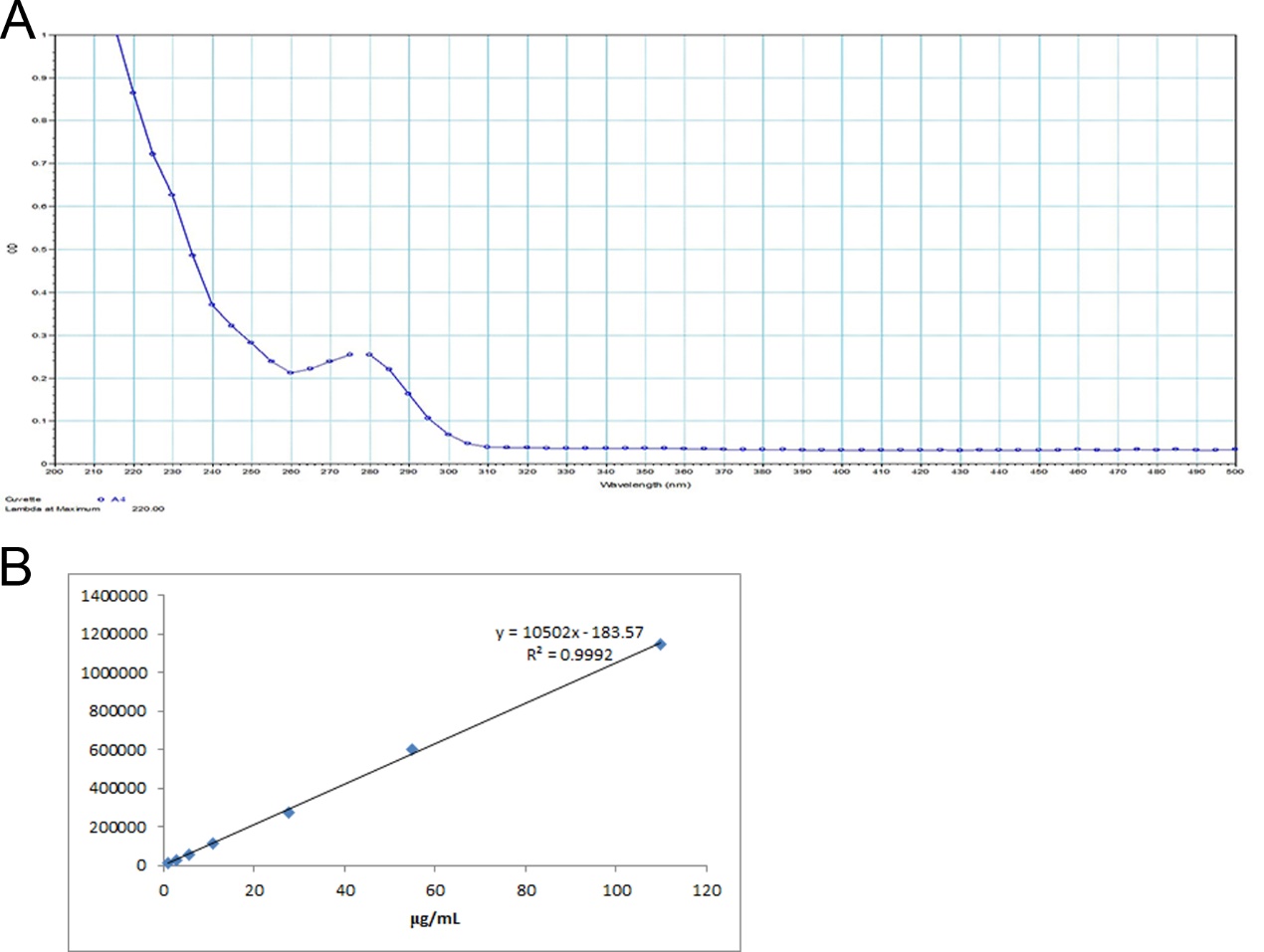


**Figure S1.** (**A**) UV obsorption spectrum of GSK-J1. (**B**) Calibration curve of GSK-J1.


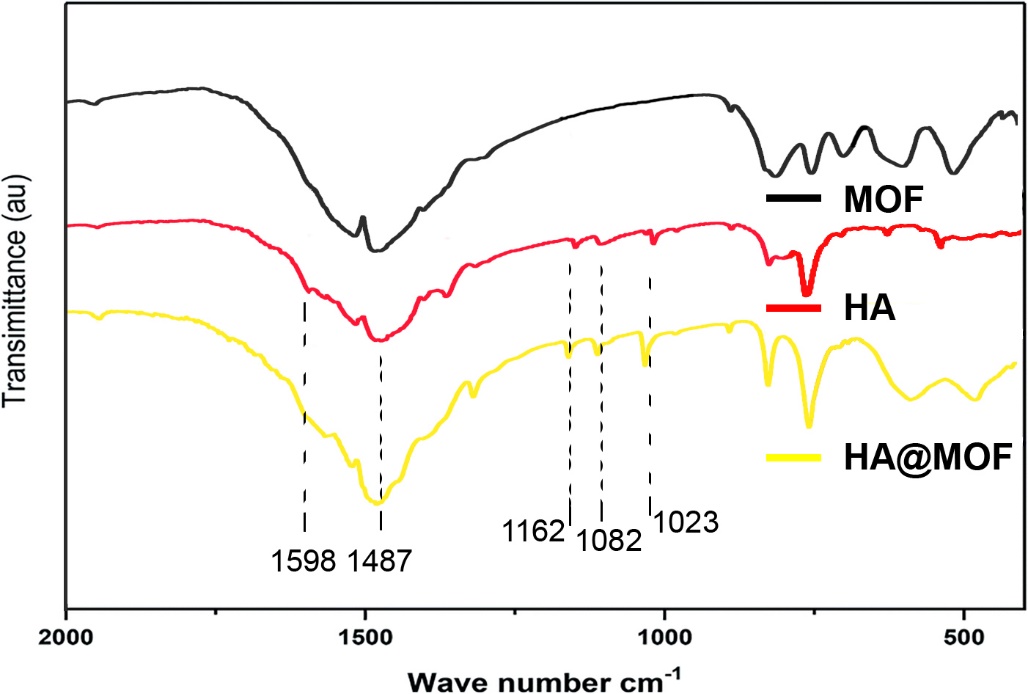


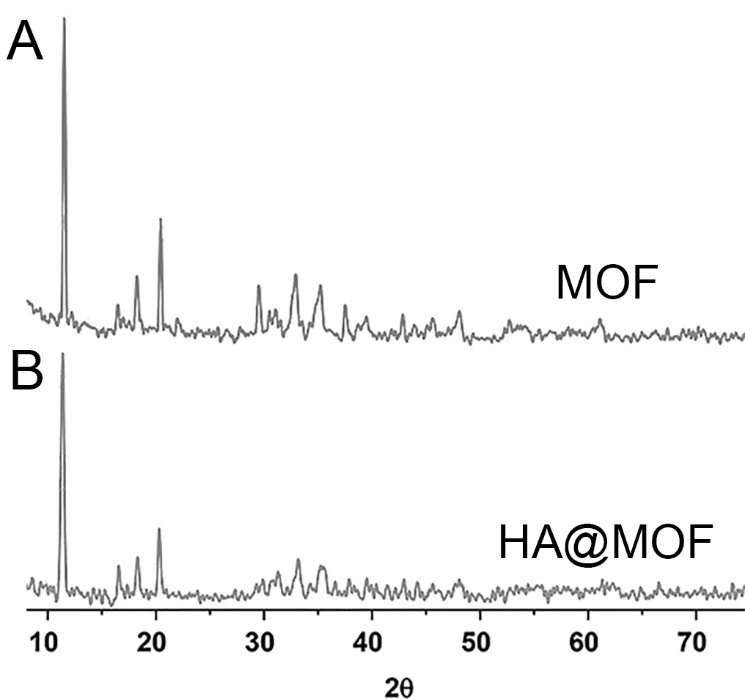


**Figure S2.** (**A**) FT-IR spectra of MOF, HA and HA@MOF. (**B**) Powder X-ray diffractions of MOF and HA@MOF.

**
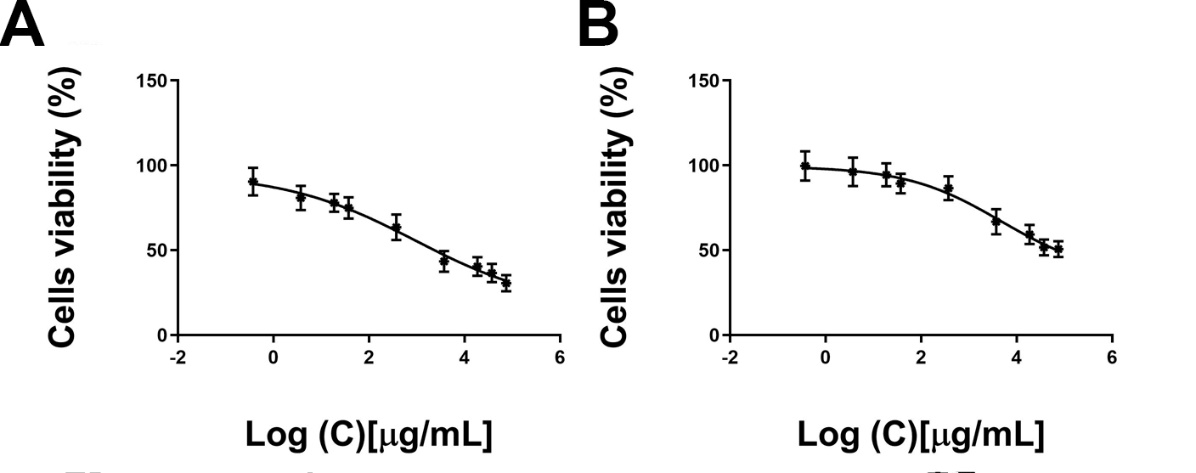
**

**Figure S3.** Dose-dependent cytotoxicity of carboplatin on (**A**) primitive SKOV-3 cells (IC_50_= 0.94 ± 0.12 μg/mL) and (**B**) carboplatin-resistant SKOV-3 cells (IC_50_= 4.70 ± 1.15 μg/mL).

**Table S1.** Primers for RT-qPCR and ChIP-qPCR used in this study.

| **Gene (human)** | **Forward** | | **reverse** |
| --- | --- | --- | --- |
| **HER2** | | **CCAGACCATAGCACACTCGG** | **GACGCCTTCAGCACGAACT** |
| **RB1** | | **TTGGATCACAGCGATACAAACTT** | **AGCGCACGCCAATAAAGACAT** |
| **GAPDH** | | **CTGGGCTACACTGAGCACC** | **AAGTGGTCGTTGAGGGCAATG** |
| **JMJD3** | | **CGCTGCCTCACCCATATCC** | **ATCCGCGACCTCTGAACTCT** |
| **MYCN** | | **CACGTCCGCTCAAGAGTGTC** | **GTTTCTGCGACGCTCACTGT** |
| **E2F1** | | **CATCCCAGGAGGTCACTTCTG** | **GACAACAGCGGTTCTTGCTC** |
| **EZH2** | | **AATCAGAGTACATGCGACTGAGA** | **GCTGTATCCTTCGCTGTTTCC** |
| **HER2 (ChIP-qPCR)** | | **CGGGAGCAAGACAAATGG** | **GCAGTGCGAAGAGCAGAAC** |
| **MYCN (ChIP-qPCR)** | | **CCCTGCTATTTTGCACCTT** | **GACCGATGCTTCTAACCCA** |
